# Predictors of Hangover Frequency and Severity: The Impact of Alcohol Consumption, Mental Resilience, Personality, Lifestyle, Coping and Mood

**DOI:** 10.3390/jcm12113811

**Published:** 2023-06-01

**Authors:** Joris C. Verster, Julie A. Donders, Anne S. Boogaard, Gillian Bruce

**Affiliations:** 1 Division of Pharmacology, Utrecht Institute for Pharmaceutical Sciences, Utrecht University, 3584 CG Utrecht, The Netherlands; j.a.donders@students.uu.nl (J.A.D.); a.s.boogaard@students.uu.nl (A.S.B.); 2 Centre for Human Psychopharmacology, Swinburne University, Melbourne, VIC 3122, Australia; 3 Division of Psychology and Social Work, School of Education and Social Sciences, University of the West of Scotland, Paisley PA1 2BE, UK; gillian.bruce@uws.ac.uk

**Keywords:** mental resilience, personality, lifestyle, coping, alcohol, hangover, severity, frequency

## Abstract

Mental resilience is the ability to bounce back from daily life stressors such as divorce or losing a job. Extensive research has demonstrated a negative relationship between mental resilience and alcohol consumption. That is, both the quantity and frequency of alcohol consumption are greater in individuals with lower levels of mental resilience. There has, however, been little scientific attention paid to the relationship between mental resilience and alcohol hangover severity. The objective of this study was to evaluate psychological factors that may impact the frequency and severity of alcohol hangovers, including alcohol intake itself, mental resilience, personality, baseline mood, lifestyle, and coping mechanisms. An online survey was conducted among Dutch adults (N = 153) who had a hangover after their heaviest drinking occasion in the period before the start of the COVID-19 pandemic (15 January to 14 March 2020). Questions were asked about their alcohol consumption and hangover severity on their heaviest drinking occasion. Mental resilience was assessed with the Brief Mental Resilience scale, personality with the Eysenck Personality Questionnaire–Revised Short Scale (EPQ-RSS), mood via single item assessments, and lifestyle and coping with the modified Fantastic Lifestyle Checklist. The partial correlation, corrected for estimated peak blood alcohol concentration (BAC), between mental resilience and hangover severity was not significant (r = 0.010, *p* = 0.848). Furthermore, no significant correlations were found between hangover severity or frequency and personality and baseline mood. For lifestyle and coping factors, a negative correlation was found between the use of tobacco and toxins (i.e., drugs, medicines, caffeine) and the frequency of experiencing hangovers. Regression analysis revealed that hangover severity after the heaviest drinking occasion (31.2%) was the best predictor of hangover frequency, and that subjective intoxication on the heaviest drinking occasion (38.4%) was the best predictor of next-day hangover severity. Mood, mental resilience, and personality were not relevant predictors of hangover frequency and severity. In conclusion, mental resilience, personality, and baseline mood do not predict hangover frequency and severity.

## 1. Introduction

The alcohol hangover is defined as the combination of negative mental and physical symptoms which can be experienced after a single episode of alcohol consumption, starting when blood alcohol concentration (BAC) approaches zero [1]. Common symptoms of the hangover state are being tired, having a headache, and concentration problems [2,3,4]. Hangovers are reported at any age [5], by both sexes [6], and can occur after consuming any amount of alcohol [7]. There are, however, great interindividual differences in the frequency and severity of alcohol hangovers [7], and there is even a considerable group of drinkers that claim to be hangover-resistant (i.e., they do not experience hangovers) [8,9,10]. Research is ongoing to evaluate potential predictors of hangover frequency and severity. With regard to alcohol intake, the amount of alcohol consumed has been shown to be a poor predictor of the occurrence and severity of hangovers [7]. Instead, several studies revealed that the level of subjective intoxication was the best predictor of hangover severity [7,11,12], while hangover severity on recent drinking occasions was positively associated with hangover frequency [13]. In this study, other potential determinants of hangover frequency and severity such as mental resilience, personality, baseline mood, and lifestyle factors will be considered.

To investigate associations with hangover severity in a scientifically sound manner, it is important to evaluate only participants that actually experienced a hangover. Thus, hangover-resistant drinkers should be excluded from the analysis. In addition, partial correlations should be used rather than uncorrected Spearman’s or Pearson’s correlations. A correction for alcohol intake is essential when evaluating hangover severity. This can be achieved by computing a partial correlation, correcting for the amount of alcohol consumed that resulted in the hangover. However, a correction for the estimated peak BAC is more precise, as this also takes into account sex, body weight, and the duration of the drinking session [14]. Alternatively, regression analysis can be applied to investigate how the combination of these different factors predicts hangover frequency or severity.

Mental resilience, i.e., the ability to bounce back [15], is an important factor in health and disease [16]. Increased levels of mental resilience have shown to be a protective factor that reduces the susceptibility to disease, facilitates better coping with stress and emotion regulation in case of mood changes, and enables quicker recovery from illness [16,17,18,19]. The relationship between alcohol consumption and mental resilience has been demonstrated previously. For example, lower levels of mental resilience are reflected in poorer coping strategies and correlate with increased alcohol use [20]. However, the literature on mental resilience and alcohol hangover is scarce, and a search yielded only three studies [21,22,23]. The first study investigating mental resilience in the context of the alcohol hangover was conducted by Van Schrojenstein Lantman et al. [21]. This online survey among *n* = 2295 Dutch students found no significant differences in mental resilience between hangover-sensitive and hangover-resistant drinkers, assessed using the Brief Resilience Scale, BRS [15]. A second survey was conducted among *n* = 341 Dutch young adults reporting on their past month’s heaviest drinking occasion [22]. No significant partial correlation, corrected for estimated BAC, was found between hangover severity and mental resilience. In contrast, a third study among *n* = 90 Australian adults found a significant positive correlation between mental resilience and hangover severity [23]. There were various methodological differences between the studies by van de Loo et al. [22] and Terpstra et al. [23] that may have had an impact on the opposite outcomes of the studies, including recruiting different samples (students with a mean age of 20.9 years versus the general adult population with a mean age of 47.8 years, respectively) and using a different hangover severity assessment (a single item rating versus the Alcohol Hangover Severity Scale, respectively). However, the biggest impact might have been the methodology applied to assess the possible association between mental resilience and hangover severity. The studies conducted different partial correlation analyses. In Van de Loo et al.’s study [22] the assessment of estimated BAC and hangover severity were from the same past month’s heaviest drinking occasion. In contrast, Terpstra et al. [23] computed a partial correlation using data on hangover severity from the participant’s latest hangover occasion during the past 30 days, whereas they controlled for the number of drinks consumed on the past month’s heaviest drinking occasion. The latter occasion might have been a different day than the latest hangover and thus unrelated to the hangover severity assessment. Moreover, controlling for the number of alcoholic drinks instead of estimated BAC did not take into account important factors such as drinking duration, sex, and body weight [14]. Therefore, the results of this study must be interpreted with great caution. Taken together, there is limited evidence that mental resilience has an impact on hangover severity, but more research is needed to confirm this. In addition, none of the three studies collected data on hangover frequency and its possible relationship with mental resilience.

With regard to personality, previous research has shown psychoticism and extraversion to be associated with higher levels of alcohol consumption, whereas increased levels of neuroticism are associated with lower levels of alcohol consumption [24,25,26]. Furthermore, higher scores on socialization (social desirability) may be related to higher levels of alcohol consumption, depending on the drinking norms of peers and to what extent individuals conform to these norms [27]. Only a few studies evaluated whether personality can have an impact on the occurrence and severity of alcohol hangover.

In 1981, Harburg et al. investigated the impact of personality and mood during a drinking session on next-day hangovers in *n* = 1266 adult participants. In a first publication [28], Harburg et al. reported positive correlations between hangover symptom frequency and psychosocial factors, including negative life events, neuroticism, guilt about drinking, feeling depressed while drinking, and being angry while drinking. In a second publication [29], Harburg et al. excluded all sober subjects from their dataset and reanalyzed the data of the remaining *n* = 1104 participants. Significant correlations were reported between hangover symptom frequency and neuroticism, guilt about drinking, drinking to escape, negative life events, feelings of depression, and anger while drinking. However, stepwise linear regression analysis revealed poor models with guilt about drinking as the strongest predictor of hangover symptom frequency (9% in men and 11% in women). As discussed in detail elsewhere [11], the study had several methodological shortcomings that complicated the interpretation of the results. For example, participants without hangovers were included in the sample used for the analysis, and hangover symptom frequency was assessed rather than hangover severity. The status of the reported findings was therefore unclear.

More recently, Verster et al. [11] found no significant correlation between hangover severity and neuroticism in *n* = 313 young adults. Instead, this study found that subjective intoxication was the best predictor of hangover severity. Terpstra et al. [23] evaluated personality in *n* = 90 Australian adults using the 10-item Big Five inventory (BFI-10) [30], a 10-item scale assessing extraversion, agreeableness, conscientiousness, neuroticism, and openness. No significant correlations were found between hangover severity and scores of the personality scales.

Baseline mood has been associated with the severity and susceptibility to hangovers. For example, Piasecki et al. [31] found that experiencing depressive symptoms was associated with having a greater susceptibility to experiencing hangovers. In addition, Royle et al. [32] found that drinkers who had higher levels of pain catastrophizing reported experiencing more severe hangovers. However, when controlling for estimated BAC, Saeed et al. [33] found no significant correlations between hangover severity and frequency with sensitivity to pain or overall pain catastrophizing. In other studies, baseline mood did not predict the occurrence or severity of alcohol hangovers [11,34], and a recent semi-naturalistic study confirmed that there are no clinically meaningful differences in baseline stress, anxiety, and depression between hangover-sensitive and hangover-resistant drinkers [35].

A healthy lifestyle may contribute to better immune fitness, i.e., the capacity of the body to respond to health challenges (such as infection or excessive alcohol intake) by activating an appropriate immune response, which is essential to maintaining health, preventing and resolving disease, and improving quality of life [36]. It has been postulated that better immune fitness may reduce the chances of having hangovers. That is, studies have revealed a significant difference in baseline immune fitness between hangover-sensitive and hangover-resistant drinkers. The baseline immune fitness of the latter group was significantly better [37], and additionally, the reduction in immune fitness the day after alcohol consumption was much greater among hangover-sensitive drinkers [38]. On the other hand, van de Loo et al. [12,22] found no significant correlation between immune fitness and hangover severity in two surveys. Related to adequate immune fitness are factors that promote a healthy lifestyle, such as adequate sleep, a healthy diet, having a normal body mass index, and regular physical activity [39,40,41,42]. There is, however, limited research to what extent these factors impact the frequency and severity of alcohol hangover. In previous studies, baseline physical activity level was not significantly related to hangover severity [11,34]. Most studies have shown that sleep is impaired after alcohol consumption, and that poorer sleep quality and reduced total sleep time have been correlated with reporting more severe hangovers [43,44,45,46,47,48,49]. However, research on the impact of baseline sleep characteristics on hangover frequency and severity is currently lacking. Finally, coping factors such as the support of family and friends or the ability to cope with stress may have an impact on both drinking behavior and the frequency and severity of hangovers. In the context of alcohol hangover, these factors have not been investigated yet.

Taken together, there is limited knowledge on predictors of hangover severity and frequency. In addition, contradictory findings on the relationship between personality and hangover severity and the methodological shortcomings of some of the previous studies warrant further investigation on this topic. To better understand the pathology of the alcohol hangover, identifying predictors of hangover frequency and severity is necessary. Therefore, the purpose of the current study was to further investigate possible predictors of hangover frequency and severity. To this extent, in addition to alcohol consumption outcomes related to usual alcohol intake and the past month’s heaviest drinking occasion, relationships were investigated with mental resilience, personality, baseline mood, coping, and lifestyle factors. Based on previous research, it was hypothesized that hangover severity is the best predictor of hangover frequency and that subjective intoxication is the best predictor of hangover severity.

## 2. Materials and Methods

Data from an online survey conducted among the Dutch adult population was used for the current analysis [50]. The survey was conducted between 24 June and 26 July 2020 via SurveyMonkey, and participants were recruited via a Facebook advertisement. There were no exclusion criteria for participating. The Ethics Committee of the Faculty of Social and Behavioral Sciences at Utrecht University granted ethical approval (approval code: FETC17-061, approval date: 8 June 2017), and electronic informed consent was obtained from all participants. For the current analysis, participants were selected who had consumed alcohol before the 2019 coronavirus disease (COVID-19) pandemic and completed the questions on mental resilience and personality that were asked in Part 2 of the survey. A detailed description of the survey methodology is published elsewhere [50].

The demographic data included sex, age, and weight. Participants were asked questions about their alcohol consumption for the period before the start of the COVID-19 pandemic (15 January to 14 March 2020). Questions were adapted and modified from the Quick Drinking Screen (QDS) [51,52]. Questions on usual alcohol consumption included the number of alcoholic drinks they consumed on average per week and the number of days they consumed alcohol per week. Guidelines were given to make sure that there was consistency in the reporting of a unit of alcohol across participants. The number of hangovers experienced per month was also recorded. For the heaviest drinking occasion in the period, the number of alcoholic drinks that were consumed was recorded as well as the duration of the drinking session. The estimated blood alcohol concentration (BAC) was computed using a modified Widmark equation, taking into account the quantity of alcohol consumed, body weight, and sex of the participant [14]. Subjective intoxication was rated on a scale from 0 (absent) to 10 (extremely drunk) [53]. Next-day hangover severity was rated on a scale ranging from 0 (absent) to 10 (severe) [54].

Mental resilience was assessed using the Brief Resilience Scale (BRS) [15]. This 6-item scale measures the ability to bounce back from stressful situations. Each item is scored on a scale ranging from 1 (“strongly disagree”) to 5 (“strongly agree”), with some items reverse scored. The average score across the 6 items is the outcome of the BRS, and higher BRS scores imply better mental resilience. Cronbach’s alpha of the BRS ranged between 0.80 and 0.91 [15]. Using the Dutch translation of the BRS, significant correlations were found between mental resilience psychological coping strategies, immune fitness, and health outcomes [55,56].

Personality traits were assessed using the Dutch version of the Eysenck Personality Questionnaire–Revised Short Scale (EPQ-RSS) [57]. The 48-item questionnaire consists of 4 subscales assessing psychoticism, extraversion, neuroticism, and socialization. The scale on socialization is used to assess the level of social desirability in answering personality questions, which is used as a correcting factor when calculating partial correlations. All subscales contain 12 items that can be answered by “yes” (score 1) or “no” (score 0), with some items reverse scored. The sum of item scores ranges from 0 and 12, with higher scores implying that the participant has a higher level of the corresponding personality trait. Cronbach’s alpha of the subscales was acceptable (0.35–0.52 for psychoticism, 0.81–0.84 for neuroticism, 0.72–0.84 for extraversion, and 0.69–0.71 for socialization) [57].

Mood was assessed for the period before the start of the COVID-19 pandemic (15 January to 14 March 2020) using single item scales, including ‘stress’, ‘anxiety’, ‘depression’, ‘fatigue’, ‘hostility’, ‘loneliness’, and ‘happiness’. All items were scored on 1-item scales ranging from 0 (absent) to 10 (extreme). Quality of life was assessed on a scale ranging from 0 (very poor) to 10 (excellent). The single items were validated previously and had high test–retest reliability [58,59].

Factors of importance for coping, emotion regulation, and healthy lifestyle were assessed for the period before the start of the COVID-19 pandemic (15 January to 14 March 2020) using a modified FANTASTIC Lifestyle Checklist [60,61,62,63]. The original scale comprised 25 questions answered on a scale ranging from 0 to 4. For the purpose of this study, the FANTASTIC Lifestyle Checklist was translated, modified, and shortened to 16 items [50]. Sum-scores were computed for the domains including support of family and friends, physical activity level, nutrition, use of tobacco and toxins, and coping with stress, whereas single-item scores were used for sleep (“I sleep well and feel rested”), optimism (“I am a positive or optimistic thinker”), and role satisfaction (“I am satisfied with my job or role”). Higher scores on the scales/items implied a better or healthier lifestyle.

### Statistical Analysis

The data were recoded and transferred to IBM SPSS Statistics for Windows version 29.0, released 2013 (IBM Corp., Armonk, NY, USA). Data were checked to verify if they were reliable. Participants with a height under 1 m and weight below 40 kg were excluded from the dataset. Furthermore, only participants who were 18 years of age or older, reported alcohol consumption before the COVID-19 pandemic, and reported having a hangover after the past month’s heaviest drinking occasion were included.

Mean and SD (standard deviation) were computed for each variable. Outcomes for males and females were compared using the Independent Samples Mann–Whitney U Test. Differences were considered significant if *p* < 0.05. For all variables except personality, partial correlations were calculated between hangover severity on the heaviest drinking occasion and the study outcome, with the estimated blood alcohol concentration (BAC) on the past month’s heaviest drinking occasion as the control variable. For personality outcomes, partial correlations were computed correcting for estimated BAC and social desirability. For correlations with hangover frequency and usual alcohol consumption outcomes, Spearman’s correlations were computed. Correlations with mental resilience were considered statistically significant if *p* < 0.05. For personality, correlations were considered significant if *p* < 0.017, and for mood and lifestyle factors, the significance cut-off was set at *p* < 0.00625.

Finally, stepwise regression analysis was conducted to predict hangover frequency and severity. All variables assessed were included in the analysis, including demographics, mental resilience, personality, mood, lifestyle, and coping factors.

## 3. Results

A total of *n* = 511 participants completed the survey. Among them, 352 reported alcohol consumption in the past month and 153 participants reported a hangover after their heaviest drinking occasion. These 153 participants were included in the analysis. Their mean (SD) age was 31.9 (14.4) years (range from 18 to 78 years). The sample comprised 57 males and 96 females. The demographics and study outcomes are summarized in Table 1.

Males were older than females, they consumed significantly more alcoholic drinks per week, and they reported significantly more drinking days per week. Additionally, on the heaviest drinking occasion, males consumed more alcohol than females. Males also reported more hangovers per month than females, but the difference did not reach statistical significance due to the stringent Bonferroni’s correction. The level of psychoticism was significantly higher in men than in women. No sex differences were significant for other personality traits, mental resilience, and baseline mood. Males reported significantly better scores for coping with stress than females. No other lifestyle factors differed significantly between males and females.

### 3.1. Mental Resilience

Correlations between mental resilience and alcohol outcomes are shown in Table 2. None of the correlations between alcohol outcomes and mental resilience were significant.

### 3.2. Personality

Table 3 shows the partial correlation between alcohol outcomes and personality.

Significant positive correlations were found between the level of extraversion and the number of alcoholic drinks consumed per week, and between the level of psychoticism and the number of drinking days per week. For the heaviest drinking occasion, the level of neuroticism was negatively correlated with the number of alcoholic drinks consumed. None of the personality scales correlated significantly with other drinking variables, including hangover severity.

### 3.3. Correlates of Hangover Frequency and Severity

Correlations between possible predictors with hangover frequency and severity are shown in Table 4. Both usual alcohol consumption and alcohol outcomes on the heaviest drinking occasion were all significant predictors of hangover frequency. The positive correlations implied that increased alcohol intake (both quantity and frequency) was associated with experiencing hangovers more frequently. Hangover frequency was also positively and significantly associated with hangover severity. Drinking duration and level of subjective intoxication were also significantly correlated with hangover severity. For mood and lifestyle, none of the correlations with hangover frequency and severity were significant.

Finally, regression analysis was conducted to identify predictors of hangover frequency and severity. Stepwise regression analysis yielded models that predicted 39.9% of hangover frequency. Significant predictors of hangover frequency were hangover severity after the heaviest drinking occasion (31.2%), number of alcoholic drinks consumed per week (5.8%), number of alcoholic drinks consumed on the heaviest drinking occasion (1.8%), and support of family and friends (1.5%). Stepwise regression analysis revealed a model that predicted 48.9% of hangover severity. Significant predictors of hangover severity were subjective intoxication on the heaviest drinking occasion (38.4%), hangover frequency (9.2%), and estimated BAC on the heaviest drinking occasion (1.3%).

## 4. Discussion

In this study, no significant correlations were found between hangover severity and mental resilience or between hangover severity and personality traits, when controlling for estimated BAC and social desirability. Baseline mood had no impact on frequency and severity of alcohol hangovers, and no significant correlations were found with adopting a healthy lifestyle (e.g., good sleep, healthy food, regular exercise) or adequate coping strategies (e.g., support of family and friends or coping with stress). A negative correlation was found between the use of tobacco and toxins (i.e., drugs, medicines, caffeine) and the frequency of experiencing hangovers, but not their severity. Regression analysis revealed that hangover severity after the heaviest drinking occasion (31.2%) was the best predictor of hangover frequency, and that subjective intoxication on the heaviest drinking occasion (38.4%) was the best predictor of next-day hangover severity. Mood, mental resilience, personality, and lifestyle factors were not relevant predictors of hangover frequency and severity.

These findings were in line with previous studies that found no relationship between hangover severity and mental resilience, personality, and baseline mood. An exception was the positive and significant correlation between hangover severity and metal resilience by Terpstra et al. However, as discussed previously, it is very likely that methodological shortcomings account for this observation. Van de Loo et al. [22] and the current study found nonsignificant correlations close to zero. Although Harburg et al. [28,29] reported that personality and mood may impact hangover severity, this study also suffered from significant methodological shortcomings. Subsequent studies revealed no significant associations between personality and mood and hangover severity [11,23].

The analysis revealed significant sex differences for age, weight, alcohol consumption (quantity and frequency), psychoticism, and coping with stress. In line with common findings, males consumed more alcohol (both quantity and frequency) than females [64]. The observation that males scored significantly higher on psychoticism than females was also in line with previous findings [65]. Finally, a sex difference in coping with stress was found. The observed difference may be related to the use of different coping strategies between males and females [66] and the age difference between males and females [67]. Notwithstanding these sex differences, the regression analysis revealed that neither sex nor any of these variables were significant predictors of hangover frequency and severity.

The strengths of the current study included its sound methodology. This included using estimated BAC as a control variable for partial correlations with alcohol outcomes of the heaviest drinking occasions. Another strength was using social desirability as a control variable for partial correlations with personality traits. Participation in the study was anonymous, which should have limited the need for giving socially desirable answers. However, a limitation of the study may be the retrospective nature of the assessments, which may have introduced recall bias. The sample size was sufficient, but relatively small, and not limited to the student population. Instead, the sample include both sexes, participants with diverse backgrounds and educational levels, and a wide age range (see reference [51] for more detail on the study sample). Unfortunately, the relatively small sample size did not allow further investigation of these factors. Future studies with a larger sample size could investigate the role of demographic and socioeconomic factors on the frequency and severity of alcohol hangovers.

The current study evaluated personality, mental resilience, and mood as possible predictors of alcohol hangovers. No significant correlations were found. Instead, hangover severity after the heaviest drinking occasion was the best predictor of hangover frequency. In addition, subjective intoxication on the heaviest drinking occasion was the best predictor of next-day hangover severity. The latter was in line with previous findings [13].

It is important to note that experimental studies into the pathology of the alcohol hangover revealed additional predictors of hangover severity. Current thinking on the pathology of the alcohol hangover suggests that a quick breakdown of ethanol into acetaldehyde is associated with less severe hangovers [68,69]. In this context, significant correlations have been found between urine ethanol concentration and hangover severity [70]. No significant correlations were found between hangover severity and urine concentrations of acetaldehyde [68], methanol [71], 5-hydroxytryptophol (5-HTOL), 5-hydroxyindoleacetic acid (5-HIAA), the 5-HTOL/5-HIAA ratio [72], ethyl glucuronide (EtG), and ethyl sulfate (EtS) [73], Research further suggests that the inflammatory response to alcohol consumption is involved in the pathology of the alcohol hangover [74]. In this context, concentrations of biomarkers of systemic inflammation (e.g., cytokines) assessed in blood or saliva showed significant correlations with hangover severity [75,76]. Finally, other factors such as dietary nutrient intake [77], the consumption of fast food versus healthy food on drinking occasions [78], and the congener content of alcoholic drinks [44,79] have been associated with hangover severity. It is important that future studies continue to evaluate potential biomarkers of hangover severity. To date, hangover severity is assessed using subjective assessments via single-item scales or multiple-item questionnaires rating the severity of symptoms [54]. Biomarkers could provide an objective assessment. With regard to hangover frequency, it would be interesting to evaluate if the frequently of experiencing hangovers is related to the development of chronic systemic inflammation and an increased susceptibility to disease.

## 5. Conclusions

Mental resilience, personality, baseline mood, and lifestyle and coping had no relevant impact on hangover frequency and severity. The best predictor of hangover frequency was hangover severity. The best predictor of hangover severity was subjective intoxication.

## Figures and Tables

**Table 1:** Demographics and study outcomes.

Demographics	Overall	Males	Females	*p*-Value
N (%)	153	57	96	-
Age (year)	31.9 (14.4)	36.8 (17.0)	29.1 (11.9)	0.004 *
Weight (kg)	72.9 (14.0)	81.1 (14.4)	68.1 (11.4)	<0.001 *
Usual alcohol consumption				
Alcoholic drinks per week	7.8 (8.3)	11.0 (10.9)	5.9 (5.6)	<0.001 *
Drinking days per week	2.5 (1.6)	3.1 (1.9)	2.1 (1.3)	0.001 *
Hangover frequency per month	1.2 (1.3)	1.6 (1.5)	1.0 (1.0)	0.022
Heaviest drinking occasion				
Number of alcoholic drinks	9.3 (6.2)	12.0 (7.9)	7.7 (4.1)	<0.001 *
Drinking duration (h)	6.0 (2.6)	6.1 (3.2)	5.9 (2.3)	0.873
Subjective intoxication	5.6 (2.4)	5.6 (2.8)	5.6 (2.2)	0.994
Estimated BAC (%)	0.12 (0.1)	0.13 (0.1)	0.11 (0.1)	0.543
Hangover severity	4.2 (2.6)	4.0 (2.6)	4.4 (2.6)	0.394
Mental resilience and personality				
Mental resilience	3.5 (0.8)	3.6 (0.8)	3.4 (0.8)	0.156
Psychoticism	2.6 (2.0)	3.5 (2.0)	2.0 (1.8)	<0.001 *
Extraversion	8.3 (3.4)	8.6 (2.9)	8.1 (3.6)	0.621
Neuroticism	4.9 (3.3)	4.0 (3.1)	5.4 (3.4)	0.007
Social desirability	6.3 (2.4)	5.7 (2.3)	6.7 (2.4)	0.018
Baseline mood				
Stress	4.1 (2.5)	3.6 (2.6)	4.3 (2.5)	0.128
Anxiety	2.9 (2.7)	2.8 (2.6)	2.9 (2.8)	0.672
Depression	2.9 (2.8)	2.8 (2.9)	3.0 (2.8)	0.614
Fatigue	4.4 (2.6)	4.3 (2.8)	4.5 (2.5)	0.555
Hostility	0.9 (1.7)	1.2 (1.9)	0.8 (1.6)	0.135
Loneliness	2.1 (2.3)	2.3 (2.4)	2.0 (2.2)	0.633
Happiness	6.8 (1.7)	6.8 (1.5)	6.8 (1.9)	0.368
Quality of life	7.3 (1.6)	7.2 (1.5)	7.4 (1.6)	0.367
Lifestyle and coping				
Support of family and friends	6.1 (1.8)	5.6 (1.8)	6.4 (1.7)	0.010
Physical activity level	5.5 (1.9)	5.2 (1.9)	5.7 (1.9)	0.157
Nutrition	8.1 (2.9)	7.6 (3.0)	8.4 (2.7)	0.112
Use of tobacco and toxins	12.8 (2.3)	12.4 (2.6)	13.0 (2.1)	0.172
Sleep	2.7 (1.0)	2.8 (1.0)	2.7 (1.0)	0.704
Coping with stress	5.6 (1.7)	6.3 (1.5)	5.3 (1.7)	<0.001 *
Optimism	3.1 (0.9)	3.2 (0.7)	3.0 (1.0)	0.261
Role satisfaction	2.9 (0.9)	2.9 (1.0)	2.9 (0.9)	0.836

Mean and standard deviation (SD, between brackets) are shown. Significant differences between males and females (*p* < 0.00625, after Bonferroni’s correction for multiple comparisons) are indicated by *. Abbreviation: BAC = blood alcohol concentration.

**Table 2:** Correlations between alcohol outcomes and mental resilience.

Usual Alcohol Consumption	r	*p*-Value
Alcoholic drinks per week	0.084	0.304
Drinking days per week	0.054	0.504
Hangover frequency per month	−0.008	0.920
Heaviest drinking occasion		
Number of alcoholic drinks	0.134	0.101
Drinking duration (h)	0.100	0.218
Subjective intoxication	−0.064	0.431
Hangover severity	−0.077	0.348

For usual alcohol consumption, Spearman’s correlations are shown. For the heaviest drinking occasion, partial correlations, corrected for estimated BAC, are shown. None of the correlations were statistically significant (*p* < 0.05). Abbreviation: BAC = blood alcohol concentration.

**Table 3:** Partial correlations between alcohol outcomes and personality.

	Psychoticism		Extraversion		Neuroticism	
Usual Alcohol Consumption	R	*p*-Value	r	*p*-Value	r	*p*-Value
Alcoholic drinks per week	0.106	0.196	0.274	<0.001 *	−0.097	0.238
Drinking days per week	0.255	0.002 *	0.133	0.104	−0.109	0.183
Hangover frequency per month	0.021	0.796	0.189	0.020	−0.006	0.944
Heaviest drinking occasion						
Number of alcoholic drinks	0.157	0.056	0.180	0.028	−0.282	<0.001 *
Drinking duration (h)	−0.048	0.561	0.189	0.020	−0.136	0.098
Subjective intoxication	−0.013	0.877	0.121	0.139	0.058	0.481
Hangover severity	−0.081	0.327	0.049	0.548	0.176	0.031

For usual alcohol consumption, partial correlations are shown, corrected for social desirability. For the heaviest drinking occasion, partial correlations, corrected for estimated BAC and social desirability, are shown. Significant partial correlations (*p* < 0.017, after Bonferroni’s correction for multiple comparisons) are indicated by *.

**Table 4:** Correlates of hangover frequency and severity.

	Hangover Frequency		Hangover Severity	
Usual Alcohol Consumption	r	*p*-Value	r	*p*-Value
Alcoholic drinks per week	0.530	<0.001 *	0.152	0.070
Drinking days per week	0.317	<0.001 *	0.017	0.843
Hangover frequency per month	-	-	0.480	<0.001 *
Heaviest drinking occasion				
Number of alcoholic drinks	0.494	<0.001 *	0.075	0.372
Drinking duration (h)	0.315	<0.001 *	0.332	<0.001 *
Subjective intoxication	0.483	<0.001 *	0.510	<0.001 *
Estimated BAC (%)	0.427	<0.001 *	0.081	0.339
Hangover severity	0.480	<0.001 *	-	-
Baseline mood				
Stress	0.180	0.026	0.162	0.046
Anxiety	0.133	0.100	0.088	0.284
Depression	0.125	0.122	0.148	0.069
Fatigue	0.181	0.025	0.023	0.779
Hostility	0.062	0.448	0.051	0.535
Loneliness	0.097	0.233	0.041	0.620
Happiness	0.002	0.979	0.076	0.355
Quality of life	0.035	0.670	0.032	0.693
Lifestyle and coping				
Support of family and friends	−0.085	0.297	0.058	0.475
Physical activity level	0.112	0.166	0.140	0.086
Nutrition	−0.150	0.065	−0.040	0.627
Use of tobacco and toxins	−0.272	<0.001 *	−0.106	0.092
Sleep	−0.170	0.036	−0.038	0.646
Coping with stress	−0.056	0.491	−0.155	0.056
Optimism	−0.045	0.579	0.005	0.953
Role satisfaction	−0.021	0.796	−0.043	0.602

Spearman’s correlations are shown for hangover severity. For correlations with hangover severity on the heaviest drinking occasion, partial correlations were computed, correcting for estimated BAC. Significant correlations (*p* < 0.00625, after Bonferroni’s correction for multiple comparisons) are indicated by *.

## Data Availability

The survey and data are available upon request from the corresponding author.

## References

[1] Verster J.C., Scholey A., van de Loo A.J.A.E., Benson S., Stock A.-K. (2020). Updating the definition of the alcohol hangover. J. Clin. Med..

[2] Penning R., McKinney A., Verster J.C. (2012). Alcohol hangover symptoms and their contribution to the overall hangover severity. Alcohol Alcohol..

[3] Van Schrojenstein Lantman M., Mackus M., van de Loo A.J.A.E., Verster J.C. (2017). The impact of alcohol hangover symptoms on cognitive and physical functioning, and mood. Hum. Psychopharmacol..

[4] Mackus M., Van de Loo A.J.A.E., Van Neer R.H.P., Vermeulen S.A., Terpstra C., Brookhuis K.A., Scholey A., Verster J.C. (2023). Differences in next-day adverse effects and impact on mood of an evening of heavy alcohol consumption between hangover-sensitive drinkers and hangover-resistant drinkers. J. Clin. Med..

[5] Verster J.C., Severeijns N.R., Sips A.S.M., Saeed H.M., Benson S., Scholey A., Bruce G. (2021). Alcohol hangover across the lifespan: Impact of sex and age. Alcohol Alcohol..

[6] Van Lawick van Pabst A.E., Devenney L.E., Verster J.C. (2019). Sex differences in the presence and severity of alcohol hangover symptoms. J. Clin. Med..

[7] Verster J.C., Kruisselbrink L.D., Slot K.A., Anogeianaki A., Adams S., Alford C., Arnoldy L., Ayre E., Balikji S., Benson S. (2020). on behalf of the Alcohol Hangover Research Group. Sensitivity to experiencing alcohol hangovers: Reconsideration of the 0.11% blood alcohol concentration (BAC) threshold for having a hangover. J. Clin. Med..

[8] Howland J., Rohsenow D.J., Edwards E.M. (2008). Are some drinkers resistant to hangover? A literature review. Curr. Drug Abus. Rev..

[9] Verster J.C., de Klerk S., Bervoets A.C., Kruisselbrink D. (2013). Can Hangover Immunity be Really Claimed?. Curr. Drug Abus. Rev..

[10] Kruisselbrink L.D., Bervoets A.C., de Klerk S., van de Loo A.J.A.E., Verster J.C. (2017). Hangover resistance in a Canadian university student population. Addict. Behav. Rep..

[11] Verster J.C., Arnoldy L., van de Loo A.J.A.E., Benson S., Scholey A., Stock A.-K. (2020). The impact of mood and subjective intoxication on hangover severity. J. Clin. Med..

[12] Van de Loo A.J.A.E., Kerssemakers N., Scholey A., Garssen J., Kraneveld A.D., Verster J.C. (2020). Perceived immune fitness, individual strength, and hangover severity. Int. J. Environ. Res. Public Health.

[13] Verster J.C., Slot K.A., Arnoldy L., Van Lawick van Pabst A.E., van de Loo A.J.A.E., Benson S., Scholey A. (2019). The association between alcohol hangover frequency and severity: Evidence for reverse tolerance?. J. Clin. Med..

[14] Watson P.E., Watson I.D., Batt R.D. (1981). Prediction of blood alcohol concentrations in human subjects. Updating the Widmark Equation. J. Stud. Alcohol.

[15] Smith B., Dalen J., Wiggins K., Tooley E., Christopher P., Bernard J. (2008). The Brief Resilience Scale: Assessing the ability to bounce back. Int. J. Behav. Med..

[16] Babiü R., Babiü M., Rastoviü P., Ûurlin M., Šimiü J., Mandiü K., Pavloviü K. (2020). Resilience in health and illness. Psychiatr. Danub..

[17] Shrivastava A., Desousa A. (2016). Resilience: A psychobiological construct for psychiatric disorders. Indian J. Psychiatry..

[18] Franklin T.B., Saab B.J., Mansuy I.M. (2012). Neural mechanisms of stress resilience and vulnerability. Neuron.

[19] Bowes L., Jaffee S.R. (2013). Biology, genes, and resilience: Toward a multidisciplinary approach. Trauma Violence Abus..

[20] Sayette M.A. (2017). The effects of alcohol on emotion in social drinkers. Behav. Res. Ther..

[21] Van Schrojenstein Lantman M., Van de Loo A.J.A.E., Mackus M., Brookhuis K.A., Kraneveld A.D., Garssen J., Verster J.C. (2018). Susceptibility to alcohol hangovers: Not just a matter of being resilient. Alcohol Alcohol..

[22] Van de Loo A.J.A.E., van Schrojenstein Lantman M., Mackus M., Scholey A., Verster J.C. (2018). Impact of mental resilience and perceived immune functioning on the severity of alcohol hangover. BMC Res. Notes.

[23] Terpstra C., Verster J.C., Scholey A., Benson S. (2022). Associations between mental resilience, mood, coping, personality, and hangover severity. J. Clin. Med..

[24] Lac A., Donaldson C.D. (2019). Personality traits moderate connections from drinking attitudes to alcohol use and myopic relief, self-inflation, and excess. Subst. Use Misuse.

[25] Fairbairn C.E., Sayette M.A., Wright A.G., Levine J.M., Cohn J.F., Creswell K.G. (2015). Extraversion and the rewarding effects of alcohol in a social context. J. Abnorm. Psychol..

[26] Lac A., Donaldson C.D. (2016). Alcohol attitudes, motives, norms, and personality traits longitudinally classify nondrinkers, moderate drinkers, and binge drinkers using discriminant function analysis. Addict. Behav..

[27] Borsari B.E., Carey K.B. (1999). Understanding fraternity drinking: Five recurring themes in the literature, 1980–1998. J. Am. Coll. Health.

[28] Harburg E., Davis D., Cummings K.M., Gunn R. (1981). Negative affect, alcohol consumption and hangover symptoms among normal drinkers in a small community. J. Stud. Alcohol..

[29] Harburg E., Gunn R., Gleiberman L., DiFranceisco W., Schork A. (1993). Psychosocial factors, alcohol use, and hangover signs among social drinkers: A reappraisal. J. Clin. Epidemiol..

[30] Rammstedt B., John O.P. (2007). Measuring personality in one minute or less: A 10-item short version of the Big Five Inventory in English and German. J. Res. Personal..

[31] Piasecki T.M., Trela C.J., Mermelstein R.J. (2017). Hangover symptoms, heavy episodic drinking, and depression in young adults: A cross-lagged analysis. J. Stud. Alcohol Drugs.

[32] Royle S., Owen L., Roberts D., Marrow L. (2020). Pain catastrophising predicts alcohol hangover severity and symptoms. J. Clin. Med..

[33] Saeed H.M., Sips A.S.M., Owen L.J., Verster J.C. (2021). The relationship between pain sensitivity, pain catastrophizing, and hangover severity. Int. J. Environ. Res. Public Health.

[34] Verster J.C., Anogeianaki A., Kruisselbrink L.D., Alford C., Stock A.-K. (2020). Relationship of alcohol hangover and physical endurance performance: Walking the Samaria Gorge. J. Clin. Med..

[35] Kim A.J., Merlo A., Mackus M., Bruce G., Johnson S.J., Alford C., Sherry S.B., Stewart S.H., Verster J.C. (2023). Depression, anxiety, and stress among hangover sensitive and hangover resistant drinkers. J. Clin. Med..

[36] Verster J.C., Kraneveld A.D., Garssen J. (2023). The assessment of immune fitness. J. Clin. Med..

[37] Van de Loo A.J.A.E., Mackus M., van Schrojenstein Lantman M., Kraneveld A.D., Garssen J., Scholey A., Verster J.C. (2018). Susceptibility to alcohol hangovers: The association with self-reported immune status. Int. J. Environ. Res. Public Health.

[38] Merlo A., Mackus M., Van de Loo A.J.A.E., Bruce G., Garssen J., Verster J.C. (2023). Immune fitness the day after an evening of heavy alcohol consumption is reduced in both hangover sensitive drinkers and hangover resistant drinkers. Alcohol. Clin. Exp. Res..

[39] Besedovsky L., Lange T., Haack M. (2019). The Sleep-Immune Crosstalk in Health and Disease. Physiol. Rev..

[40] Van Oostrom E.C., Mulder K.E.W., Verheul M.C.E., Hendriksen P.A., Thijssen S., Kraneveld A.D., Vlieg-Boerstra B., Garssen J., Verster J.C. (2022). A healthier diet is associated with greater immune fitness. PharmaNutrition.

[41] Kiani P., Mulder K.E.W., Balikji J., Kraneveld A.D., Garssen J., Verster J.C. (2022). Pandemic Preparedness: Maintaining Adequate Immune Fitness by Attaining a Normal, Healthy Body Weight. J. Clin. Med..

[42] Nieman D.C., Wentz L.M. (2019). The compelling link between physical activity and the body’s defense system. J. Sport Health Sci..

[43] Rohsenow D.J., Howland J., Minsky S.J., Arnedt J.T. (2006). Effects of heavy drinking by maritime academy cadets on hangover, perceived sleep, and next-day ship power plant operation. J. Stud. Alcohol.

[44] Rohsenow D.J., Howland J., Arnedt J.T., Almeida A.B., Greece J., Minsky S., Kempler C.S., Sales S. (2010). Intoxication with bourbon versus vodka: Effects on hangover, sleep, and next-day neurocognitive performance in young adults. Alcohol Clin. Exp. Res..

[45] Rohsenow D.J., Howland J., Alvarez L., Nelson K., Langlois B., Verster J.C., Sherrard H., Arnedt J.T. (2014). Effects of caffeinated vs. non-caffeinated alcoholic beverage on next-day hangover incidence and severity, perceived sleep quality, and alertness. Addict. Behav..

[46] Van Schrojenstein Lantman M., Mackus M., Roth T., Verster J.C. (2017). Total sleep time, alcohol consumption and the duration and severity of alcohol hangover. Nat. Sci. Sleep.

[47] Van Schrojenstein Lantman M., Roth T., Roehrs T., Verster J.C. (2017). Alcohol hangover, sleep quality, and daytime sleepiness. Sleep Vigil..

[48] Devenney L.E., Coyle K.B., Roth T., Verster J.C. (2019). Sleep after heavy alcohol consumption and physical activity levels during alcohol hangover. J. Clin. Med..

[49] Ayre E., Scholey A., White D., Devilly G., Kaufman J., Verster J.C., Allen C., Benson S. (2021). The relationship between alcohol hangover severity, sleep and cognitive performance; a naturalistic study. J. Clin. Med..

[50] Kiani P., Merlo A., Saeed H.M., Benson S., Bruce G., Hoorn R., Kraneveld A.D., Severeijns N.R., Sips A.S.M., Scholey A. (2021). Immune fitness, and the psychosocial and health consequences of the COVID-19 pandemic lockdown in The Netherlands: Methodology and design of the CLOFIT study. Eur. J. Investig. Health Psychol. Educ..

[51] Dum M., Sobell L.C., Sobell M.B., Heinecke N., Voluse A., Ohnson K. (2009). A Quick Drinking Screen for identifying women at risk for an alcohol-exposed pregnancy. Addict. Behav..

[52] De Haan L., de Haan H., Olivier B., Verster J.C. (2012). Alcohol mixed with energy drinks: Methodology and design of the Utrecht Student Survey. Int. J. Gen. Med..

[53] Benson S., Verster J.C., Alford C., Scholey A. (2014). Effects of mixing alcohol with caffeinated beverages on subjective intoxication: A critical review and meta-analysis. Neurosci. Biobehav. Rev..

[54] Verster J.C., van de Loo A.J.A.E., Benson S., Scholey A., Stock A.-K. (2020). The assessment of overall hangover severity. J. Clin. Med..

[55] Van Schrojenstein Lantman M., Otten L.S., Mackus M., de Kruijff D., van de Loo A.J.A.E., Kraneveld A.D., Garssen J., Verster J.C. (2017). Mental resilience, perceived immune functioning, and health. J. Multidiscip. Healthc..

[56] Balikji J., Hoogbergen M.M., Garssen J., Verster J.C. (2022). Mental resilience, mood, and quality of life in young adults with self-reported impaired wound healing. Int. J. Environ. Res. Public Health.

[57] Sanderman R., Arrindell W.A., Ranchor A.V., Eysenck H.J., Eysenck S.B.G. (2012). Het Meten van Persoonlijkheidskenmerken Met de Eysenck Personality Questionnaire (EPQ), Een Handleiding.

[58] Verster J.C., Sandalova E., Garssen J., Bruce G. (2021). The use of single-item ratings versus traditional multiple-item questionnaires to assess mood and health. Eur. J. Investig. Health Psycho. Educ..

[59] Verster J.C., Mulder K.E.W., Hendriksen P.A., Verheul M.C.E., van Oostrom E.C., Scholey A., Garssen J. (2023). Test-retest reliability of single-item assessments of immune fitness, mood and quality of life. Heliyon.

[60] Wilson D.M.C., Ciliska D. (1984). Development and use of the FANTASTIC checklist. Can. Fam. Physician.

[61] Sharratt J.K., Sharratt M.T., Smith D.M., Howell N.J., Davenport L. (1984). FANTASTIC Lifestyle survey of University of Waterloo employees. Can. Fam. Physician.

[62] Wilson D.M.C., Nielsen E., Ciliska D. (1984). Lifestyle assessment: Testing the FANTASTIC Instrument. Can. Fam. Physician.

[63] Canadian Society for Exercise Physiology Fantastic Lifestyle Checklist. https://rowingbc.ca/wp-content/uploads/2016/12/Fantastic-Lifestyle-Checklist.pdf.

[64] Slade T., Chapman C., Swift W., Keyes K., Tonks Z., Teesson M. (2016). Birth cohort trends in the global epidemiology of alcohol use and alcohol-related harms in men and women: Systematic review and metaregression. BMJ Open.

[65] Lynn R., Martin T. (1997). Gender differences in extraversion, neuroticism, and psychoticism in 37 nations. J. Soc. Psychol..

[66] Graves B.S., Hall M.E., Dias-Karch C., Haischer M.H., Apter C. (2021). Gender differences in perceived stress and coping among college students. PLoS ONE.

[67] Folkman S., Lazarus R.S., Pimley S., Novacek J. (1987). Age differences in stress and coping processes. Psychol. Aging.

[68] Mackus M., van de Loo A.J.A.E., Garssen J., Kraneveld A.D., Scholey A.D., Verster J.C. (2020). The role of alcohol metabolism in the pathology of alcohol hangover. J. Clin. Med..

[69] Mackus M., van de Loo A.J.A.E., Garssen J., Kraneveld A.D., Scholey A., Verster J.C. (2020). The association between ethanol elimination rate and hangover severity. Int. J. Environ. Res. Public Health.

[70] Van de Loo A.J.A.E., Mackus M., Korte-Bouws G.A.H., Brookhuis K.A., Garssen J., Verster J.C. (2017). Urine ethanol concentration and alcohol hangover severity. Psychopharmacology.

[71] Mackus M., van de Loo A.J.A.E., Korte-Bouws G.A.H., van Neer R.H.P., Wang X., Nguyen T.T., Brookhuis K.A., Garssen J., Verster J.C. (2017). Urine methanol concentration and alcohol hangover severity. Alcohol.

[72] Mackus M., van de Loo A.J.A.E., van den Boogaard W.J.M., Korte-Bouws G.A.H., Verster J.C. (2021). The 5HTOL/5HIAA ratio as a biomarker of the alcohol hangover. J. Clin. Med..

[73] Mackus M., van de Loo A.J.A.E., Raasveld S.J., Hogewoning A., Sastre Toraño J., Flesch F.M., Korte-Bouws G.A.H., van Neer R.H.P., Wang X., Nguyen T.T. (2017). Biomarkers of the alcohol hangover state: Ethyl glucuronide (EtG) and ethyl sulfate (EtS). Hum. Psychopharmacol..

[74] Van de Loo A.J.A.E., Mackus M., Kwon O., Krishnakumar I., Garssen J., Kraneveld A.D., Scholey A., Verster J.C. (2020). The inflammatory response to alcohol consumption and its role in the pathology of alcohol hangover. J. Clin. Med..

[75] Kim D.J., Kim W., Yoon S.J., Choi B.M., Kim J.S., Go H.J., Kim Y.K., Jeong J. (2003). Effects of alcohol hangover on cytokine production in healthy subjects. Alcohol.

[76] Van de Loo A.J.A.E., Raasveld S.J., Hogewoning A., de Zeeuw R., Bosma E.R., Bouwmeester N.H., Lukkes M., Knipping K., Mackus M., Kraneveld A.D. (2021). Immune responses after heavy alcohol consumption: Cytokine concentrations in hangover sensitive and hangover resistant drinkers. Healthcare.

[77] Verster J.C., Vermeulen S.A., van de Loo A.J.A.E., Balikji S., Kraneveld A.D., Garssen J., Scholey A. (2019). Dietary nutrient intake, alcohol metabolism, and hangover severity. J. Clin. Med..

[78] Kösem Z., van de Loo A.J.A.E., Fernstrand A.M., Garssen J., Verster J.C. (2015). The impact of consuming food or drinking water on alcohol hangover. Eur. Neuropsychopharmacol..

[79] Verster J.C. (2006). Congeners and alcohol hangover: Differences in severity among Dutch college students after consuming beer, wine or liquor. Alcohol. Clin. Exp. Res..

